# Evaluating Applicant Perceptions of the Impact of Social Media on the 2020-2021 Residency Application Cycle Occurring During the COVID-19 Pandemic: Survey Study

**DOI:** 10.2196/29486

**Published:** 2021-10-05

**Authors:** Ariana Naaseh, Sean Thompson, Steven Tohmasi, Warren Wiechmann, Shannon Toohey, Alisa Wray, Megan Boysen-Osborn

**Affiliations:** 1 University of California, Irvine School of Medicine Irvine, CA United States

**Keywords:** residency application, social media, medical education, resident, medical student, perspective, residency recruitment, virtual application, virtual residency

## Abstract

**Background:**

Due to challenges related to the COVID-19 pandemic, residency programs in the United States conducted virtual interviews during the 2020-2021 application season. As a result, programs and applicants may have relied more heavily on social media–based communication and dissemination of information.

**Objective:**

We sought to determine social media’s impact on residency applicants during an entirely virtual application cycle.

**Methods:**

An anonymous electronic survey was distributed to 465 eligible 2021 Match applicants at 4 University of California Schools of Medicine in the United States.

**Results:**

A total of 72 participants (15.5% of eligible respondents), applying to 16 specialties, responded. Of those who responded, 53% (n=38) reported following prospective residency accounts on social media, and 89% (n=34) of those respondents were positively or negatively influenced by these accounts. The top three digital methods by which applicants sought information about residency programs included the program website, digital conversations with residents and fellows of that program, and Instagram. Among respondents, 53% (n=38) attended virtual information sessions for prospective programs. A minority of applicants (n=19, 26%) adjusted the number of programs they applied to based on information found on social media, with most (n=14, 74%) increasing the number of programs to which they applied. Survey respondents ranked social media’s effectiveness in allowing applicants to learn about programs at 6.7 (SD 2.1) on a visual analogue scale from 1-10. Most applicants (n=61, 86%) felt that programs should use social media in future application cycles even if they are nonvirtual.

**Conclusions:**

Social media appears to be an important tool for resident recruitment. Future studies should seek more information on its effect on later parts of the application cycle and the Match.

## Introduction

In the United States, the residency selection process begins when fourth-year medical students (US seniors) submit their residency applications through the Electronic Residency Application Service. It ends on “Match Day,” when the National Resident Matching Program informs applicants of the specialty and program to which they matched. Students who match into urology and ophthalmology follow a similar process, but match through the American Urological Association residency match and San Francisco Match, respectively. The process lasts approximately six months, during which US seniors apply to an average of 70 programs [1] and rank 13 programs [2]. In 2020, 93.7% of US seniors matched to a residency position [3]. Applicants spend October through January of the application cycle traveling to and interviewing with an average of 13 programs [2]. In selecting a residency program, applicants consider many factors, including program location, reputation, “fit,” curriculum quality, work/life balance, quality of faculty, and program size, among others [2]. Traditionally, residency programs have relied heavily on interview day to introduce and recruit applicants to their program. As social media and digital influence become more integrated into global culture, there has been a paradigm shift in residency recruitment methods. Many US residency programs across several specialties have expanded their websites and increased their social media presence [4-7].

Due to the COVID-19 pandemic, the Coalition for Physician Accountability recommended that all interviews during the 2020-2021 application season be conducted virtually [8]. As such, programs were limited in their ability to interact with and recruit candidates due to the virtual nature of the interview season and virtual methods of connection became even more important.

Few studies have surveyed how residency applicants are influenced by social media, with none doing so across all specialties. It has been previously shown that social media plays a moderate role in influencing anesthesia applicants' decisions of which programs to apply to and rank highly [9]. However, prior studies focused primarily on a single social media platform, such as Facebook [10]. With the COVID-19 pandemic and the transition to a virtual interview process, medical students were faced with the challenge of obtaining information through nontraditional means.

Our objective was to explore the extent of applicants’ use of social media during an entirely virtual residency match cycle. We also sought to determine whether decisions regarding where to apply were influenced by social media.

## Methods

In October 2020, we surveyed 2021 US residency applicants via an anonymous REDCap-generated survey [11]. Identifying information, such as school attended, was not collected to maintain the privacy of respondents. The survey, detailed in full in Multimedia Appendix 1, was distributed via listserv email to medical students at the University of California (UC) Irvine, UC Davis, UC San Francisco, and UC Riverside. In total, 465 students were eligible to complete the survey across the 4 sites. Students were sent reminder emails via their respective listserv once every 2 weeks over a 6-week time frame to encourage survey participation. This study was exempt from Institutional Review Board approval by UC because participants were anonymous and the study posed minimal risk of ascertaining participant identities.

## Results

Of the 465 students who were emailed the survey, 72 (15.5%) completed the survey and represented multiple specialties (Table 1). Applicants applying to more than one specialty selected all applicable specialties.

**Table 1:** Overview of survey respondents by specialty.

Specialty	Students, n (%)
Anesthesia	6 (8.3)
Dermatology	1 (1.4)
Emergency medicine	5 (6.9)
Family medicine	12 (16.7)
General surgery	9 (12.5)
Integrated surgery (cardiothoracic, vascular)	1 (1.4)
Internal medicine	11 (15.3)
Obstetrics and gynecology	4 (5.6)
Ophthalmology	4 (5.6)
Orthopedic surgery	3 (4.2)
Pediatrics	9 (12.5)
Plastic surgery	1 (1.4)
Physical medicine and rehabilitation	1 (1.4)
Psychiatry	4 (5.6)
Radiology	1 (1.4)
Urology	4 (5.6)

Of respondents, 85% (n=64) used social media prior to the 2021 residency cycle. Of those who were previously using social media, platforms used were as follows: 93% used Facebook (n=57), 85% used Instagram (n=52), 36% used YouTube (n=22), 34% used LinkedIn (n=21), 28% used Twitter (n=17), 7% used TikTok (n=4), and 2% answered Other (n=1).

**Figure 1:**
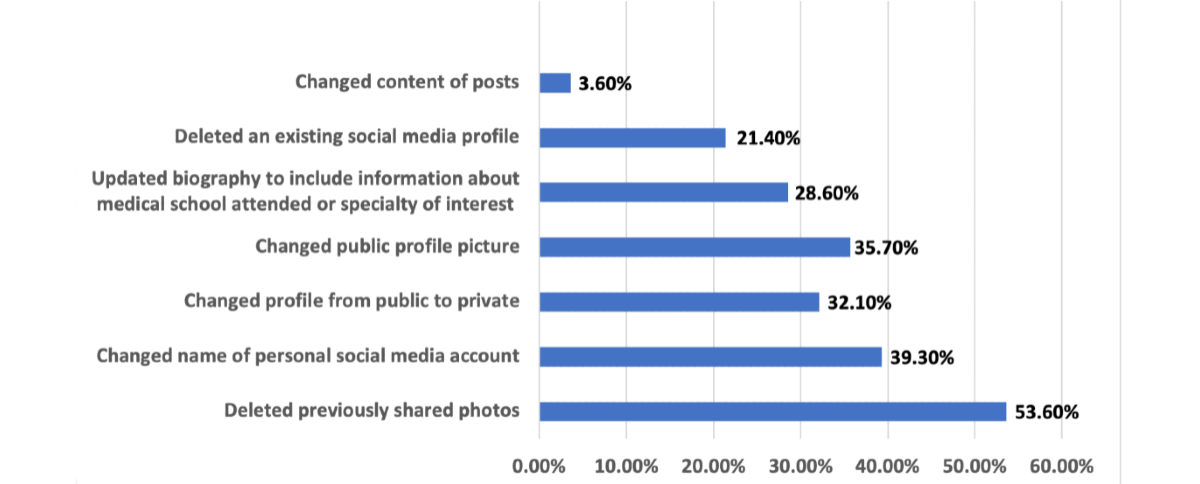
Actions performed by the 28 survey participants who reported adjusting their social media accounts prior to the residency application cycle.

Of applicants using social media prior to the residency cycle, 39% (n=28) adjusted their profiles prior to submitting applications. Actions taken by applicants are depicted in Figure 1. For those that made changes (n=28), the top reasons cited were “I wanted to avoid being portrayed in an ‘unprofessional’ light” (n=23, 82%), “I wanted to be less visible to residency programs” (n=16, 57%), “I wanted to decrease time on social media” (n=5, 18%), “I wanted to be more visible to residency programs” (n=3, 11%), and “I want to highlight present rather than past interests” (n=2, 7%).

Some applicants (n=7, 10%) created new social media accounts for the residency cycle, specifically Twitter (n=6) and Instagram (n=1).

Many respondents reported “following” specific prospective programs (n=38, 54%) or faculty, resident, or staff members of those programs (n=21, 29%) on social media. Of these, most reported being influenced (positively or negatively) in their decision to apply to a specific program based on the account (n=14, 70% for specific accounts; n=34, 89% for residency accounts).

The most common digital methods used by applicants to learn about programs were the program website, digital conversations with trainees of that prospective program, and Instagram. Other notable resources were speaking to current program faculty and residents about other prospective programs, virtual (live or recorded) information sessions, and online resources such as FREIDA and Doximity. Most respondents (n=32, 53%) indicated they attended a virtual information session.

A portion of applicants (n=19, 26%) adjusted the number of programs to which they applied based on new information found on social media platforms. Of these, most (n=14, 74%) increased the number of programs to which they applied.

On average, survey respondents ranked social media’s effectiveness in allowing applicants to learn about programs at 6.7 (SD 2.1) on a visual analogue scale, with 1 being least effective and 10 being most effective. Most students (n=61, 86%) felt that residencies should continue to use social media as a method to spread information about their program in future cycles, even if interviews revert back to an in-person format.

## Discussion

### Principal Findings

Residency applicants in 2021 at select UC medical schools in the United States used social media to interact with and learn about residency programs across a broad range of specialties. This study demonstrates that social media is a valid and necessary use of residency programs’ time and efforts to aid with recruitment of prospective trainees.

Having access to digital information may help mitigate the barriers applicants face in receiving information about prospective programs during the COVID-19 pandemic. As no prior studies have assessed how applicants learn about residency programs, it is plausible that—due to this year’s virtual application process and travel restrictions—speaking to trainees from both the applicant’s home and prospective programs became more critical than in prior application cycles.

We noticed that virtual information sessions became more prevalent during the 2020-2021 application cycle; these were attended by a majority of our survey respondents. It would be of interest to directly study how virtual residency information sessions affect applicants’ views of a program. However, it can be inferred that because a majority of applicants adjusted the number of programs applied to favorably based on social media, that increased dissemination of information can contribute to applicants applying to programs they had not considered prior to the session.

Social media can have a critical impact on the application process because it not only enables applicants to receive information about programs, but also provides programs with information about applicants. Largely, survey respondents indicated the changes they made to their profiles were to avoid being portrayed in an unprofessional light and be less visible to residency programs. However, there were a few applicants who created profiles to become more visible to residency programs. It has previously been found that program directors use social media to gain additional information about applicants [12-14]. It is possible that the applicant-specific information publicly displayed on social media may have the potential to negatively affect an applicant’s final ranking. It remains controversial whether or not it is ethical for programs to consider an applicant’s publicly available social media profiles when scoring an applicant during committee meetings for many reasons including the potential to bias their view of the applicant [15].

Additional large-scale studies are needed to fully determine if applicants are using social media as a way to display their personal hobbies, interests, or academic achievements to residency programs. A small-scale study focused on obstetrics and gynecology applicants at Brown University found a low incidence of inappropriate content or posts on applicants’ social media and postulated that this could either be a reflection of the professionalism of their applicants or their ability to hide inappropriate content [16]. Additionally, it would be valuable to assess if program directors are using social media profiles to learn about applicants and to what extent they feel this affects the preinterview, postinterview, and ranking process.

### Limitations

Limitations of this study include small sample size, somewhat low response rate of survey audience, and regional focus of surveyed applicants. In particular, the response rate could introduce bias as those more likely to complete the survey may have used social media more heavily than those who chose not to respond. However, this is the first study of its kind to look at multiple specialties. Further studies should include a more widespread survey with a larger sample size to better generalize the utility of social media. If a larger number of applicants per specialty can be achieved in a nationwide survey, then it can be deciphered which specialties are using social media more than others to engage with applicants. It would also be beneficial to stratify applicants using social media by their respective specialties to identify whether there is an association between residency program engagement online and applicant social media use.

Our survey was distributed in October 2020, after the submission of all residency applications. Therefore, our study only offers the perspectives of applicants leading up to interview season. Although understanding how applicants create their initial first impression of programs is essential, it would also be beneficial to understand how social media can influence applicant perspectives throughout interview season and through the ranking process. Understanding how programs engage with applicants throughout the entire residency application process can inform the creation of national guidelines and consensus statements regarding best conduct by both programs and applicants to limit potential Match violations.

### Conclusions

Applicants participating in the 2021 US residency application cycle believe that social media is a powerful tool for resident recruitment that should be used in future cycles. Notably, programs should focus recruitment efforts on the program website, Facebook, and Instagram, which are the top three platforms used by applicants for residency information.

## Appendix

Multimedia Appendix 1 Survey distributed to 2021 residency applicants.

## Abbreviations

UC: University of California

## References

[1] ERAS Statistics Data. Association of American Medical Colleges.

[2] Results of the 2019 NRMP Applicant Survey by Preferred Specialty and Applicant Type. National Resident Matching Program.

[3] Charting Outcomes in the Match: Senior Students of U.S. MD Medical Schools. National Resident Matching Program.

[4] Atia A, Langdell H, Hollins A, Shammas RL, Glener A, Marks C, Lee BT, Phillips BT (2021). Microsurgery Fellowship Website and Social Media Presence: Are Programs Optimizing Recruitment Strategy?. J Reconstr Microsurg.

[5] Ruddell J, Tang O, Persaud B, Eltorai A, Daniels A, Ng T (2021). Thoracic surgery program websites: Bridging the content gap for improved applicant recruitment. J Thorac Cardiovasc Surg.

[6] Mecham JC, Menapace DC, Bowe SN, Carlson ML (2021). Recruitment and Networking With Social Media for the Otolaryngology Match in the COVID-19 Pandemic. Otolaryngol Head Neck Surg.

[7] Choinski K, Carnevale M, Koleilat I, Phair J (2020). The Prevalence and Utility of Vascular Surgery Training Programs' and Vascular Societies' Social Media Presence. Ann Vasc Surg.

[8] The Coalition for Physician Accountability’s Work Group on Medical Students in the Class of 2021 Moving Across Institutions for Post Graduate Training Final Report and Recommendations for Medical Education Institutions of LCME-Accredited, U.S. Osteopathic, and Non-U.S. Medical School Applicants. Association of American Medical Colleges.

[9] Renew JR, Ladlie B, Gorlin A, Long T (2019). The Impact of Social Media on Anesthesia Resident Recruitment. J Educ Perioper Med.

[10] McHugh SM, Shaffer EG, Cormican DS, Beaman ST, Forte PJ, Metro DG (2014). Use of social media resources by applicants during the residency selection process. JEPM.

[11] Harris PA, Taylor R, Thielke R, Payne J, Gonzalez N, Conde JG (2009). Research electronic data capture (REDCap)--a metadata-driven methodology and workflow process for providing translational research informatics support. J Biomed Inform.

[12] Go P, Klaassen Z, Chamberlain R (2012). Residency selection: do the perceptions of US programme directors and applicants match?. Med Educ.

[13] Go PH, Klaassen Z, Chamberlain RS (2012). Attitudes and practices of surgery residency program directors toward the use of social networking profiles to select residency candidates: a nationwide survey analysis. J Surg Educ.

[14] Langenfeld SJ, Vargo DJ, Schenarts PJ (2016). Balancing Privacy and Professionalism: A Survey of General Surgery Program Directors on Social Media and Surgical Education. J Surg Educ.

[15] Wells D (2015). When Faced With Facebook: What Role Should Social Media Play in Selecting Residents?. J Grad Med Educ.

[16] Sullivan ME, Frishman GN, Vrees RA (2017). Showing your public face: does screening social media assess residency applicants' professionalism?. Am J Obstet Gynecol.

