# Supplementary material for: Evaluating Applicant Perceptions of the Impact of Social Media on the 2020-2021 Residency Application Cycle Occurring During the COVID-19 Pandemic: Survey Study
Source: JMIR Med Educ. 2021 Oct 5;7(4):e29486. doi: 10.2196/29486 (PMC8527380; doi:10.2196/29486)
Supplement: Multimedia Appendix 1 [file mededu_v7i4e29486_app1.pdf]

2021 Residency Match Social Media Survey

Please complete this survey so that we can best understand the role social media is playing in the 2021 residency application cycle and future cycles.

Please mark which specialty (or specialties) you are applying into during the 2021 Match cycle:

- ☐ Anesthesia
- ☐ Dermatology
- ☐ Emergency Medicine
- ☐ ENT
- ☐ Family Medicine
- ☐ General Surgery
- ☐ Integrated Surgery residency (Vascular, Cardiothoracic, etc.)
- ☐ Internal Medicine
- ☐ Interventional Radiology
- ☐ OB/GYN
- ☐ Ophthalmology
- ☐ Orthopedic Surgery
- ☐ Pathology
- ☐ Pediatrics
- ☐ Plastic Surgery
- ☐ PM&R
- ☐ Psychiatry
- ☐ Radiology
- ☐ Urology
- ☐ Other

Other:

____

Were you previously using social media prior to the virtual residency app cycle?

- ○ Yes
- ○ No

If so, which platforms were you utilizing?

- ☐ Facebook
- ☐ Instagram
- ☐ Twitter
- ☐ Youtube
- ☐ LinkedIn
- ☐ TikTok
- ☐ Tumblr
- ☐ Weibo
- ☐ Other

(Please check all that apply.)

Did you adjust your current social media profiles in any way for the residency application cycle?

- ○ Yes
- ○ No

If you adjusted your social media, how did you do so?

- ☐ Hid or deleted old photos
- ☐ Change profile photo
- ☐ Add information about medical school you are attending and/or specialty applying into bio
- ☐ Changed name or handle
- ☐ Change profile from public to private
- ☐ Change profile from private to public
- ☐ Changed content of posts
- ☐ Removed a social media profile from the internet
- ☐ Other

(Please check all that apply.)

Other:	__________________________
If you have changed content of posts, how have you done so?	☐ Limiting "unprofessional" content<br>☐ Posted extracurricular content in hopes of discussing in future interviews<br>☐ Posted content to highlight interests outside of medicine<br>☐ Posted more medicine/specialty-specific content<br>☐ Other<br>(Please check all that apply.)
If you made changes to your social media profile, why did you do so?	☐ I wanted to be less visible to residency programs<br>☐ I wanted to be more visible to residency programs<br>☐ I wanted to decrease time spent on social media<br>☐ I found myself with more free time to spend on social media<br>☐ I wanted to avoid being portrayed in an "unprofessional" light<br>☐ I wanted to highlight current rather than past interests<br>☐ Other<br>(Please check all that apply.)
Other:	__________________________
Did you create any new social media accounts for the residency cycle?	◯ Yes<br>◯ No
If so, which did you create?	☐ Facebook<br>☐ Instagram<br>☐ Twitter<br>☐ Youtube<br>☐ LinkedIn<br>☐ Tumblr<br>☐ TikTok<br>☐ Weibo<br>☐ Other<br>(Please check all that apply.)
Other:	__________________________
During this application cycle, have you followed specific faculty members/staff members/residents of prospective programs on social media?	◯ Yes<br>◯ No
Have you been positively or negatively influenced about a program by a specific faculty member/staff member/resident social media account that is not linked to the official residency program's social media account?	◯ Yes<br>◯ No
During this application cycle, have you followed official or unofficial department residency accounts of prospective programs on social media?	◯ Yes<br>◯ No

Have you been positively or negatively influenced about a program by an unofficial or official department social media account?

◯ Yes
◯ No

Check the top 3 platforms that you have utilized to learn about residency programs.

	Top Platform	2nd Platform	3rd Platform
Program website	◯	◯	◯
Speaking to residents/faculty/fellows of that program	◯	◯	◯
Speaking to residents/faculty/fellows at your home program about other programs	◯	◯	◯
Online resources such as FREIDA/Texas STAR/Doximity, etc.	◯	◯	◯
YouTube	◯	◯	◯
Virtual information sessions (real-time or pre-recorded)	◯	◯	◯
Instagram	◯	◯	◯
TikTok	◯	◯	◯
Twitter	◯	◯	◯
Facebook	◯	◯	◯
Other not listed above	◯	◯	◯

Have you attended a virtual information session or sub-internship due to information you found on social media?

◯ Yes
◯ No

Have you adjusted the number of programs you are applying to throughout the application cycle due to new information you have found on social media?

◯ Yes
◯ No

I've increased the number of programs I'm applying to due to social media.

◯ True
◯ False

I've decreased the number of programs I'm applying to due to social media.

◯ True
◯ False

How effective do you think social media platforms are in allowing prospective students to get to know residency programs?

Not effective | Neutral | Very effective

(Place a mark on the scale above)

Do you think that programs should continue to utilize social media in future application cycles even if they revert back to non-virtual interview formats?

◯ Yes ◯ No

Thank you for your time!
